# Evaluation of Regional Pulmonary Ventilation in Spontaneously Breathing Patients with Idiopathic Pulmonary Fibrosis (IPF) Employing Electrical Impedance Tomography (EIT): A Pilot Study from the European IPF Registry (eurIPFreg)

**DOI:** 10.3390/jcm10020192

**Published:** 2021-01-07

**Authors:** Ekaterina Krauss, Daniel van der Beck, Isabel Schmalz, Jochen Wilhelm, Silke Tello, Ruth C. Dartsch, Poornima Mahavadi, Martina Korfei, Eckhard Teschner, Werner Seeger, Andreas Guenther

**Affiliations:** 1 Member of the German Center for Lung Research (DZL), Universities of Giessen and Marburg Lung Center (UGMLC), Klinikstr. 33, 35392 Giessen, Germany; ekaterina.krauss@innere.med.uni-giessen.de (E.K.); daniel.v.beck@innere.med.uni-giessen.de (D.v.d.B.); isabel.schmalz@icloud.com (I.S.); jochen.wilhelm@chemie.bio.uni-giessen.de (J.W.); silke.tello@innere.med.uni-giessen.de (S.T.); ruth.dartsch@innere.med.uni-giessen.de (R.C.D.); poornima.mahavadi@innere.med.uni-giessen.de (P.M.); martina.korfei@neuro.med.uni-giessen.de (M.K.); werner.seeger@innere.med.uni-giessen.de (W.S.); 2 European IPF Registry & Biobank (eurIPFreg), 35392 Giessen, Germany; 3 Institute of Lung Health (ILH), 35392 Giessen, Germany; 4 Cardio-Pulmonary Institute (CPI) Klinikstr. 33, 35392 Giessen, Germany; 5 Draegerwerk AG & Co. KGaA, 23558 Luebeck, Germany; eckhard.teschner@draeger.com; 6 Agaplesion Lung Clinic Waldhof-Elgershausen, 35753 Greifenstein, Germany

**Keywords:** idiopathic pulmonary fibrosis (IPF), electrical impedance tomography (EIT), European Registry for idiopathic pulmonary fibrosis (eurIPFreg), interstitial lung diseases (ILD)

## Abstract

Objectives: In idiopathic pulmonary fibrosis (IPF), alterations in the pulmonary surfactant system result in an increased alveolar surface tension and favor repetitive alveolar collapse. This study aimed to assess the usefulness of electrical impedance tomography (EIT) in characterization of regional ventilation in IPF. Materials and methods: We investigated 17 patients with IPF and 15 healthy controls from the University of Giessen and Marburg Lung Center (UGMLC), Germany, for differences in the following EIT parameters: distribution of ventilation (TID), global inhomogeneity index (GI), regional impedance differences through the delta of end-expiratory lung impedance (dEELI), differences in surface of ventilated area (SURF), as well as center of ventilation (CG) and intratidal gas distribution (ITV). These parameters were assessed under spontaneous breathing and following a predefined escalation protocol of the positive end-expiratory pressure (PEEP), applied through a face mask by an intensive care respirator (EVITA, Draeger, Germany). Results: Individual slopes of dEELI over the PEEP increment protocol were found to be highly significantly increased in both groups (*p* < 0.001) but were not found to be significantly different between groups. Similarly, dTID slopes were increasing in response to PEEP, but this did not reach statistical significance within or between groups. Individual breathing patterns were very heterogeneous. There were no relevant differences of SURF, GI or CGVD over the PEEP escalation range. A correlation of dEELI to FVC, BMI, age, or weight did not forward significant results. Conclusions: In this study, we did see a significant increase in dEELI and a non-significant increase in dTID in IPF patients as well as in healthy controls in response to an increase of PEEP under spontaneous breathing. We propose the combined measurements of EIT and lung function to assess regional lung ventilation in spontaneously breathing subjects.

## 1. Introduction

Idiopathic pulmonary fibrosis (IPF) is a complex disease process affecting all compartments of the lower respiratory system, from the conducting airways to the lung vasculature. It is associated with a significant reduction in quality of life and life expectancy in general; in absence of treatment, survival ranges between 3 and 5 years from the time of diagnosis [1]. Additionally, the familial form of IPF may show a more aggressive natural course as compared to the sporadic form [2].

Although a number of risk factors have been identified, such as smoking, inhalation of toxic fumes, as well as genetic mutations or polymorphisms, the precise origin of progressive epithelial cell damage remains unclear, and unfortunately, there are still no reliable diagnostic or prognostic biomarkers [3]. Key pathophysiological features of IPF include changes in lung mechanics, such as reduced lung compliance and lung volumes, impaired gas exchange, and alterations in pulmonary haemodynamics [4].

Lung fibrosis results in a profound reduction of lung compliance. Such loss of compliance may be driven by both tissue scarring and an increase in alveolar surface tension by alterations in the pulmonary surfactant system [5]. Whereas the contribution of increased surface tension to the loss of pulmonary compliance is evident in animal models of lung fibrosis, it is currently unclear to which extent this happens in clinical IPF [6,7,8]. Especially affected are type II alveolar epithelial cells, which usually synthesize, secrete, and recycle all components of surfactant [9]. IPF lungs are characterized by a marked loss of surface tension reducing properties of surfactant, which is known to significantly correlate with the degree of lung restriction [5]. Such an impairment of surface activity is largely caused by changes in the phospholipid profile and by a loss of mature (and hence functionally active) surfactant proteins (SP) SP-B and SP-C, suggestive of an altered type II cell metabolism. A still valid hypothesis for the decline in pulmonary function and gas exchange in fibrosis is the so-called “collapse induration”, which means irreversible fusion of alveolar basement membranes in collapsed alveolar units due to increased alveolar surface tension [10].

Chest high-resolution computed tomography (HRCT) is the primary modality used in the initial assessment of patients with suspected IPF and has considerable influence on subsequent management decisions [11]. The main role of HRCT is to distinguish chronic fibrosing lung diseases with a usual interstitial pneumonia (UIP) pattern from those presenting with a non-usual interstitial pneumonia pattern, suggestive of an alternative diagnosis. UIP pattern on chest tomography is characterized by the presence of subpleural and basal predominance, reticulation, honeycombing and traction bronchiectasis, together with the absence of features suggestive of an alternative diagnosis [12].

Electrical-Impedance Tomography (EIT) is a non-invasive, radiation-free, imaging technique that relies on measuring impedance changes. The first publication of an EIT image of the human thorax was made in 1985 by Brown et al. [13]. Since then, multiple advances have been made and nowadays allow for the monitoring of lung ventilation and the assessment of regional lung function at the bedside, especially in patients with acute respiratory distress syndrome (ARDS) [14,15]. EIT allows measurement of regional ventilation based on thoracic electrical conductivity, thus evaluating the electrical properties of lung tissue and their regional variation depending on changes in air volume [16].

EIT probes the chest by rotating application of very small alternating electrical currents and measures the resulting electrical voltages [17]. A change in local air volume, for instance, its increase during inspiration or alveolar recruitment, is associated with an elongation of pathways the current needs to pass through and this result in higher values of measured electrical bioimpedance. However, local fluid content also makes a contribution to regional pulmonary electrical bioimpedance. For instance, the accumulation of fluid in the lung tissue, as in lung oedema or in the pleural space, as in empyema or pleural effusion, can be detected by EIT as a reduction in bioimpedance [17].

The EIT technique therefore provides valuable information with regard to lung aeration or ventilation, and hence lung volumes, regional over-distention and atelectasis, and homogeneity of gas distribution on a breath-by-breath basis [18]. The method can be used to create dynamic cross-sectional images of the thorax, enabling radiation-free, non-invasive monitoring of regional lung ventilation at the bedside [17]. Although the clinical interpretation of EIT images has not been standardized so far, this technology is gaining clinical importance, especially in the acute phase of respiratory failure and has been addressed in the recent European Respiratory Society (ERS) statement on chest imaging [19,20].

## 2. Aim of the Study

To the best of our knowledge, this is the first study aiming to evaluate EIT as a diagnostic, non-invasive tool to assess regional pulmonary ventilation, to estimate lung volume, over-distention and atelectasis, and to analyze homogeneity of gas distribution in IPF patients as compared to healthy controls (HC). Following a predefined protocol of PEEP increment, we also asked for recruitment of presumably collapsed lung regions.

## 3. Materials and Methods

### 3.1. Patients’ Enrollment and Data Collection

In this explorative study taking place from August to September 2013, we prospectively analyzed 32 consecutive patients recruited at the University of Giessen and Marburg Lung Center (UGMLC) into the eurIPFreg; of them were 17 patients with incident or prevalent IPF from our outpatient center for interstitial lung diseases (ILD) and 15 healthy controls (HC), taken as a comparator group. The HC group consisted mostly of patients’ relatives, who accompanied them to the doctors’ visits. The term “healthy” was self-reported and meant absence of relevant chronic diseases and absence of abnormalities by physical examination. The HC also underwent measurement of lung function, which needed to be normal.

### 3.2. EurIPFreg

The European IPF Registry (eurIPFreg) is a multicentre registry linked to the European IPF Biobank (eurIPFbank). Both, eurIPFreg and eurIPFbank are listed in ClinicalTrials.gov (NCT02951416) [21] and were permitted by institutional review boards in Germany and Europe (e.g., Ethics Committee of Justus-Liebig-University of Giessen; 111/08 and 82/13). This observatory, registry-based EIT study was separately reviewed and accepted by the Ethics Committee of Justus-Liebig-University of Giessen (No. 82/13). The research was conducted strictly according to the principles of the Declaration of Helsinki.

IPF patients were enrolled into this research if they were at least 18 years old, able to provide written informed consent for the eurIPFreg and if IPF had been diagnosed by the expert site. The diagnosis of IPF was based on the ATS guideline 2011 [22,23]. The exclusion criteria for eurIPFreg were age under 18 years, missing informed content, and pregnancy. Also, as per other EIT study protocols, patients with implanted devices with unknown compatibility to EIT, large chest wounds, and chest tubes or non-conductive bandages were excluded from this study.

### 3.3. EIT Measurements

In order to visualize regional lung ventilation in real-time and ventilatory impairments in the cohort, we used an EIT device from Draeger (PulmoVista 500, Draeger, Luebeck, Germany). The electrode belt consisted of 16 electrodes and was positioned circumferentially around the thorax at the level of the 4th to 6th intercostal space (= region of interest, ROI). During the measurement, small alternating electrical currents were delivered and measured by all electrodes. We used electrode belts of various lengths, but same number of electrodes, pending on the patient’s chest circumference. Next, a mouth/nose mask (as used for non-invasive ventilation) was applied and connected to the EVITA 4 mechanical ventilator (Draeger). The EVITA was put in Continuous Positive Airway Pressure (CPAP) mode with a PEEP level of 0 cm H_2_O (and no additional inspiratory pressure support). This allowed for measuring tidal volumes and to applying an end-expiratory PEEP.

During the measurements, the seating study subjects (IPF patients, HC) were first instructed to breathe calmly and evenly to record the tidal breathing for about 3 min. Afterwards, they were asked to perform an FRC maneuver analogous to a lung function test, i.e., a full breathing maneuver from the deep inspiration to the complete expiration. This maneuver was carried out a total of 3 times, with interim short periods of respiratory breathing. In the next phase, a stepwise increase in the end-expiratory pressure (positive end-expiratory pressure = PEEP) took place. For this purpose, the PEEP was increased stepwise in 1 cm H_2_O steps from 0 cm H_2_O to 10 cm H_2_O. At each stage, there was tidal breathing for about 60 s. After 1 min respiration at a PEEP of 10 cm H_2_O, the PEEP was down-regulated again to 0 cm H_2_O and the measurement and the data recording set were completed. The patients were instructed to stop the measurement at any time if they felt uncomfortable.

### 3.4. EIT Data Analysis and Measured Variables

Raw impedance data were used for generation of cross-sectional impedance images of the lungs, similar to computed tomography (CT) [17,18]. For our study, the EIT data were analyzed off-line using the Matlab analysis tool EITdiag and Microsoft Excel (Microsoft, Seattle, WA, USA). The end of inspiration and expiration were detected from the global impedance waveform using the software’s breath detection algorithm. The relative impedance values were calculated for every time point and image pixel as the difference between the current impedance and the baseline impedance, divided by the baseline impedance. The resulting pixel values were displayed as a tidal variation image. For data analysis, the tidal images were divided into four layers (ventral, medioventral, mediodorsal, dorsal) of equal size, comprising 32 × 8 image pixels each. The EIT plethysmogram is a waveform derived from the sum of all pixels within a given ROI of a relative image (frame) plotted against time. The global (whole image) plethysmogram has a high correlation with volume waveforms, and thus can be considered as such [24]. It represents the amount of air that moves in and out of the region of interest (ROI) [25]. In our study, we analyzed 4 different ROIs, namely a ventral, a dorsal, a medio-ventral, and a medio-dorsal. EIT data analysis was based on the EIT waveforms that are generated from a series of raw EIT images in individual image pixels. The waveform over time displays changes in local electrical impedance resulting from various physiological or pathological effects. Periodic signal fluctuations are induced by ventilation, but also by heart action and lung perfusion [17].

Image reconstruction is the process of generating raw EIT images from the measured voltages, typically of a two-dimensional slice through the electrode plane. An ideal reconstruction algorithm should guarantee uniform amplitude response, small and uniform position error, small ringing artefacts, uniform resolution, limited shape deformation, high resolution, and exhibit small sensitivity to electrode and boundary movement. As with other established examination procedures, such as CT, the EIT images are displayed caudo-cranially, with the right chest side on the left side of the image and with anterior at the top. The ROI are (from top to the bottom) ventral, mid-ventral, mid-dorsal and dorsal. As known, the inflation of the lung regions (ventral, dorsal) is often heterogeneous. The measured impedance changes correlate directly with changes in pulmonary ventilation [26]. The ventilation map or functional image is a representation of the tidal changes in impedance pixel by pixel. By positioning horizontal and/or vertical cursors in this functional image, it is possible to quantify the distribution of ventilation in the right-to-left direction, the ventral-to-dorsal direction, or to quadrants [27].

In our study, the following EIT parameters were measured either for the entire lung (“global analysis”, given as a value) or for defined areas of the EI tomogram:

- Intratidal gas distribution (ITV) exhibits the way the gas distributes in the lungs during the tidal breath and delineates the changes in regional ventilation over a time course during one breath in different ROIs.
- Tidal Impedance Distribution (TID) reflects the average ventilation for a defined period of breath, as reflected by the difference in impedance values at the beginning and the end of an inspiration. For illustration of regional changes in TID, impedance changes above 10% of the maximum regional impedance change are displayed in dark blue. As values increase, the dark blue turns into a lighter blue. A white color indicates the regions of maximum regional impedance change.
- Difference of TID over time (dTID) displays a global change of TID between two points of time, i.e., between baseline (PEEP = 0 cm H_2_O) and the various PEEP increment time points. For illustration of regional changes in dTID, the differential image indicates increases in dTID (vs. the reference) in turquoise and decreases in orange color.
- Surface of ventilated areas (SURF) describes the surface of ventilated areas of the tomogram, as defined by a regional impedance chance between inspiration and expiration. For illustration of regional changes in the tomogram, ventilated pixels are displayed in white color; non-aerated pixels are given in a dark grey color.
- Global inhomogeneity index (GI) represents the spatial extent and dispersion in the distribution of tidal breath, i.e., global inhomogeneity in tidal ventilation [28]. A GI with a value 0 represents a perfectly homogeneous distribution. For illustration of regional changes in the tomogram, the absolute differences between the median impedance value of all pixels and each single pixel value are depicted.
- Center of ventilation (CGVD) represents a vertical shift of the ventilation distribution along a gravitational axis in supine position [17]. CGVD (Center of Gravity of Ventilation Distribution) describes how ventilation is distributed between ventral and dorsal lung regions [29]. For the global analysis, this variable is expressed as a percentage of impedance on the dorsal-ventral scale from 0% (= ventilation completely in the dorsal image line) to 100% (in the ventral image line).
- End-expiratory Lung Impedance (EELI) reflects the impedance at end-expiration. The higher the EELI, the more lung volume is present at end-expiration. As the absolute lung volume is rarely known, EELI cannot be directly related to the end-expiratory lung volume.
- Difference of EELI over time (dEELI) depicts change of EELI between two points of time, i.e., the change in EELI between PEEP = 0 cm H_2_O and the various PEEP increment time points. For illustration of regional changes, the dEELI value of each pixel is indicated by a blue to white color and the more color the higher the percental increase.

The detailed description of EIT variables is offered in Supplementary Material S1.

### 3.5. Quality of Data and Statistical Analysis

As per protocol analysis, for the evaluation of the EIT data the software Draeger PV500 DataAnalysis (Draegerwerk AG & Co. KGaA, 23558 Luebeck, Germany) and Matlab Tool EITdiag (MathWorks, Natick, MA, USA) by the Draeger company were used. Using the Draeger PV500 DataAnalysis, the data and values recorded with the Pulmovista 500 could be viewed on the PC. Through the selection of specific lung areas, different ROIs could be set, for example, to contrast the ventilation of the central lung or the periphery. The Matlab EITdiag tool was used to calculate additional parameters with a wide variety of setting options. In tables, data from quantitative variables are summarized as mean ± SD and range; data from categorical variables are summarized as absolute and relative frequencies (%).

The changes over PEEP levels were analyzed by linear mixed models including the PEEP and PEEP—group interaction as fixed factors and a random slope patient. The models did not include an intercept term, as by definition dTID and dEELI are zero at PEEP = 0 cm H_2_O. *P*-values were obtained from likelihood ratio tests. These analyses were performed using R 4.0 and the packages lme4 1.1–23 and lmerTest 3.1–2 [R, lme4, lmerTest].

## 4. Results

### 4.1. Demographics and Clinical Characteristic of the Study Cohort

In this pilot study, we included 32 subjects, 17 of which were consecutive patients with IPF and 15 were HC. Table 1 displays demographics and clinical parameters including the medication of the cohort in 2013.

Our control subjects were younger compared to the patients and were characterized by normal results of lung function, diffusion capacity and capillary blood gases. In contrast, the IPF population was characterized by a moderate restrictive lung function pattern and a severe impairment in DLco and corresponding limitations in gas exchange, with 3 (17.65%) patients being on oxygen (long term oxygen supplement) with 1, 4, and 5 O_2_ L/min, respectively, as presented in Table 2.

### 4.2. Analysis of EIT Variables

In general, all patients and all HC adhered to the study protocol. Due to profound variabilities in spontaneous breathing, especially in the IPF group, inconsistent variations were frequently observed in some parameters, which somewhat limited the overall conclusiveness. Also, not all IPF patients did manage to complete the protocol until the highest PEEP level of 10. In detail, some patients interrupted the measurement at a PEEP of 8 (*n* = 1) or 9 (*n* = 10) cm H_2_O. These patients were reporting that they felt uncomfortable and would experience the end-expiratory pressure as too high. Because of this observation, we only took the data until a PEEP level of 8 cm H_2_O into account. One patient interrupted the measurement at a PEEP of 2 cm H_2_O and, thus, was omitted from the statistical analysis.

**Figure 1:**
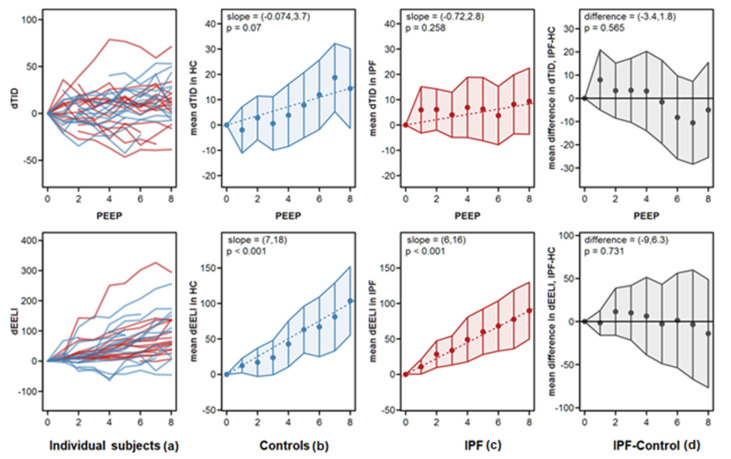
Global change in difference of tidal impedance distribution over time (dTID) and difference of end-expiratory lung impedance over time (dEELI) during PEEP titration. Panels show, from left to right: (**a**) patient’s individual tra-jectories, (**b**) mean values by PEEP in controls, (**c**) mean values by PEEP in IPF patients, and (**d**) mean differences between IPF patients and controls. Blue lines indicate data from controls; red lines indicate data from IPF patients. Points represent mean values, error bars represent pointwise 95% confidence intervals. The dotted lines are the model fits for the change over PEEP. The 95% confidence intervals of the slopes of these lines are indicated in the panels. The difference in the right panels is the 95% confidence interval of the difference between the slopes for IPF patients and controls. Abbreviations: TID—tidal impedance distribution; dTID—difference of TID over time; dEELI—difference of end-expiratory lung im-pedance over time; PEEP—positive end-expiratory pressure. The statistical analysis showed that dEELI significantly increased over the PEEP increment (*p* < 0.001).

Our measurements showed an increase in dTID and dEELI values for both groups over the period of the PEEP increment, suggestive of an improved alveolar/bronchial ventilation or recruitment of lung regions (*p* < 0.001). Within each group, the dTID increase over the PEEP protocol was not statistically significant whereas the dEELI increase was. The results are shown in Supplementary Table S1 and in Figure S1, taking the entire course of the data for each group into account. Contrary to our expectation, we, however, did not see a meaningful difference between the IPF and the HC group. As presented in Figure 1, right panel, the IPF group showed a comparable increase in dEELI and dTID over the PEEP increment.

**Figure 2:**
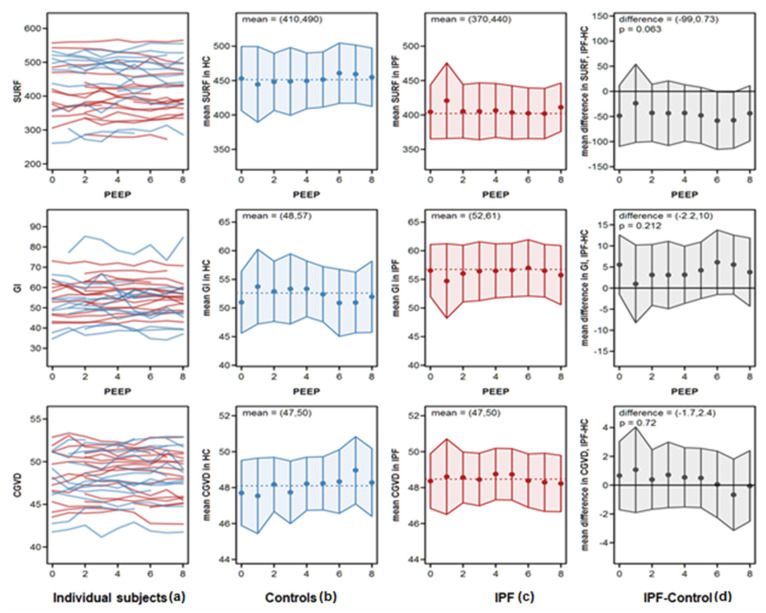
Global change in surface of ventilated areas (SURF), global inhomogeneity index (GI) and center of ventilation (CGVD) during PEEP titration. Panels show, from left to right: (**a**) patient’s individual trajectories, (**b**) mean values by PEEP in controls, (**c**) mean values by PEEP in IPF patients, and (**d**) mean differences between IPF patients and controls. Blue lines indicate data from controls; red lines indicate data from IPF patients. Points are mean values; error bars are point-wise 95% confidence intervals. The dotted lines are the model fits for the change over PEEP. The 95% confidence intervals of the slopes of these lines are indicated in the panels. The difference in the right panels is the 95% confidence interval of the difference between the slopes for IPF patients and controls.

With regard to the regional changes in ventilation, we saw a very inhomogeneous ventilation distribution in the IPF group, as reflected by the lower (albeit insignificant) SURF values in the IPF versus the HC group (Supplementary Table S1 and Figure S2). GI or CGVD were not found to be significantly different between IPF and HC, neither did these parameters change over time and in response to a PEEP increment. The data are presented in Figure 2.

In order to better approximate the individual PEEP response, we additionally performed individual slope analysis for each EIT variable. These slopes included all values measured over the PEEP increment. The results are depicted in Supplementary Figure S1 and are similar to the data shown in Figure 1 and Figure 2. The only parameters being significantly increased were dTID in IPF and dEELI in IPF and controls. Furthermore, we performed a linear regression analysis of the dEELI slope of the entire population (IPF and HC), as well as of the IPF and HC groups with age, FVC, BMI and body weight as co-variables (Supplementary Figures S2–S4). There was no dependence of the dEELI increase on age or FVC. In contrast, we observed at first a significant negative correlation of dEELI with body weight and BMI in the IPF, but not the HC group (Supplementary Figure S3, right panel). Nevertheless, such correlation could be explained by weight/gender difference, due to lower weight and higher dEELI values in women. By further adjustment of this analysis (slope “dEELI vs weight—by gender”, padj = 0.216), the effect was not significant.

### 4.3. Examples of EIT Measurements in IPF Patients

**Figure 3:**
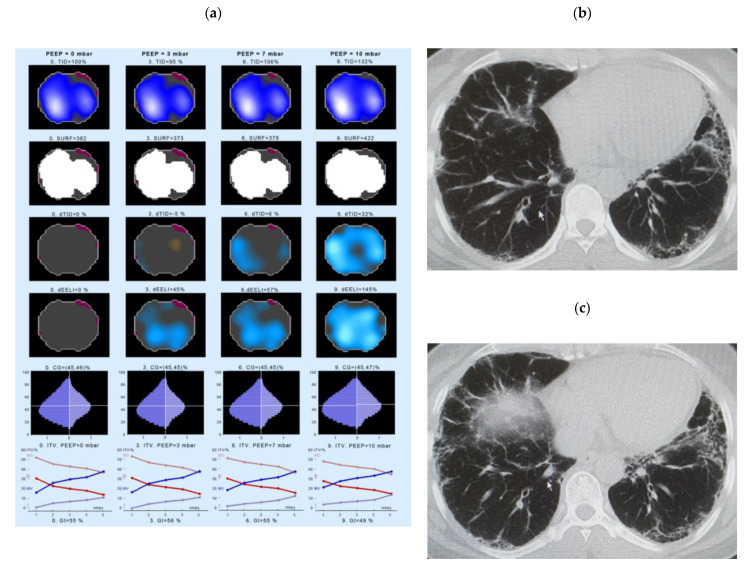
Changes in regional lung ventilation in IPF patient #4. Left panel (**a**): Spatial distribution of TID, SURF, dTID, dEELI in the electrical impedance (EI) tomogram at a PEEP of 0 (left), 3 (2nd left), 7 (3rd from left), and 10 cm H_2_O levels (right column). The lowest panel indicates the intratidal gas distribution (ITV) for four anatomical areas during one respiration. Given are the distribution of ventilation into the ventral (V), dorsal (D), medio-ventral (MV) and medio-dorsal (MD) regions of the lung. The X scale (1–5) shows the entire inspiration, divided into 5 steps. Right panel (**b**,**c**): The respective high resolution CT picture corresponding to the ROI of EIT measurement is shown. Abbreviations: CG—center of gravity of ventilation distribution; dEELI—difference of end-expiratory lung impedance over time; ITV—intratidal gas distribution; PEEP—positive end-expiratory pressure; SURF—surface of ventilated areas, TID—tidal impedance distribution; dTID—difference of TID over time.

A first example of an EIT measurement shows data of a 44-year-old woman with IPF, ex-smoker, receiving antifibrotic therapy (pirfenidone) from November 2010 until July 2011, the therapy was later paused due to the side effects. At the time of the EIT measurement the FVC was 79.9% pred., the DLco 46.9% pred. EIT and HRCT data are displayed in Figure 3.

The patient showed an increase of the thoracic volume in response to the PEEP escalation, with a steady increase in the ventilation of the medio-ventral (MV) and ventral (V) ROIs, but a decrease of inflation of the medio-dorsal (MD) and dorsal (D) areas (Figure 3, left panel, bottom line). The biggest change can be seen in the first and last 20% of the inspiration phase. At the same time, this patient showed a clear continuous increase in the change in end-expiratory lung impedance (dEELI) from 10% increase in PEEP 1 cm H_2_O to 145% at a PEEP level of 9 cm H_2_O.

**Figure 4:**
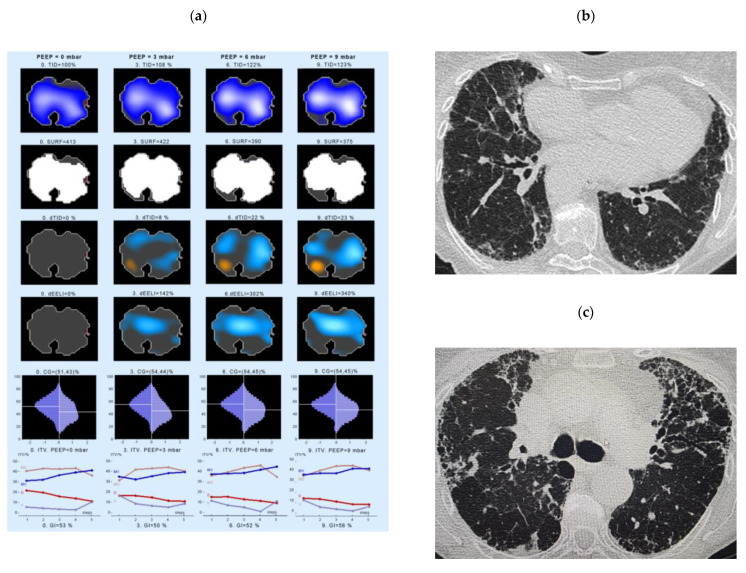
Changes in regional lung ventilation under increasing PEEP in IPF patient #14. Left panel (**a**): Spatial distribution of TID, SURF, dTID, dEELI in the EI tomogram at a PEEP of 0 (left), 3 (2nd left), 6 (3rd from left) and 9 levels (right column). The lowest panel indicates the intratidal gas distribution (ITV) for four anatomical areas during one respiration. Given are the distribution of ventilation into the ventral (V), dorsal (D), medio-ventral (MV) and medio-dorsal (MD) regions of the lung. The X scale (1–5) shows the entire inspiration, divided into 5 steps. Right panel (**b**,**c**): The respective high resolution CT picture corresponding to the ROI of EIT measurement is shown. Abbreviations: CG—center of gravity of ventilation distribution; dEELI—difference of end-expiratory lung impedance over time; ITV—intratidal gas distribution, PEEP—positive end-expiratory pressure; SURF—surface of ventilated areas; TID—tidal impedance distribution; dTID—difference of TID over time.

The second example of an EIT measurement shows a 72-year-old, non-smoker IPF patient (Figure 4). At the time of the EIT measurement, the PFT showed a FVC of 67.2% pred., DLco of 33.8 % pred. EIT shows a ventral decrease in ventilation in the first phase of inspiration. In the last section of inspiration, however, the gas distribution increases ventrally again. In the dorsal lung area, the gas distribution continuously decreases slightly during the inspiration (Figure 4, left panel, lower line). This patient showed an increase in end-expiratory lung volume in response to the PEEP application: dEELI ranged of 142% of baseline already at a PEEP level of 3 cm H_2_O and further increased to 340% at a PEEP level of 9 cm H_2_O, with the worst prominent increase in the ventilation taking place in the center and left ventral parts of the lung. The increased ventilation in ventral areas can also be seen when looking at the ITV variable (Figure 4, lowest panel), forward an increase in the ventral and the medio-ventral parts.

**Figure 5:**
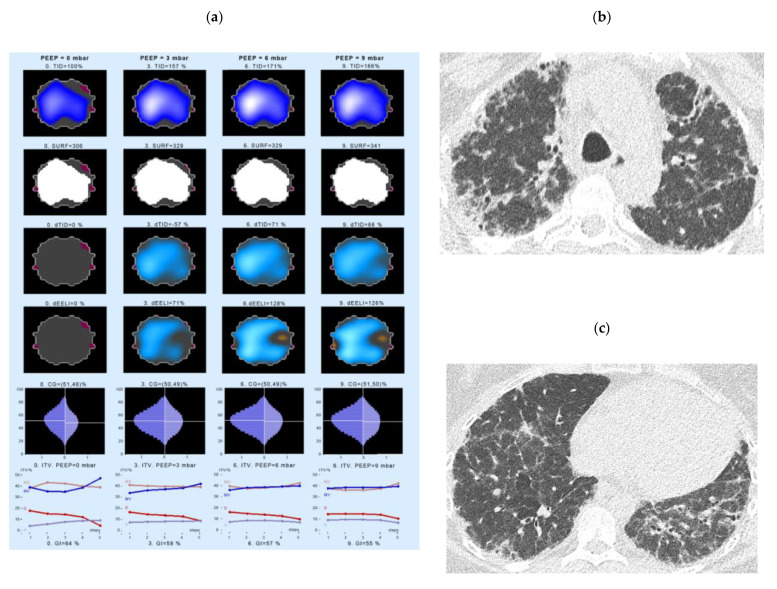
Changes in regional lung ventilation under increasing PEEP in IPF patient #8. Left panel (**a**): Spatial distribution of TID, SURF, dTID, dEELI in the EI tomogram at a PEEP of 0 (left), 3 (2nd left), 6 (3rd from left) and 9 levels (right column). The lowest panel indicates the intratidal gas distribution (ITV) for four anatomical areas during one respiration. Given are the distribution of ventilation into the ventral (V), dorsal (D), medio-ventral (MV) and medio-dorsal (MD) regions of the lung. The X scale (1–5) shows the entire inspiration, divided into 5 steps. Right panel (**b**,**c**): The respective high resolution CT picture corresponding to the ROI of EIT measurement is shown. Abbreviations: CG—center of gravity of ventilation distribution; dEELI—difference of end-expiratory lung impedance over time; ITV—intratidal gas distribution; PEEP—positive end-expiratory pressure; SURF—surface of ventilated areas; TID—tidal impedance distribution; dTID—difference of TID over time.

Another example shows the EIT measurements of a 46-year-old IPF patient #8 (Figure 5), who had never smoked in her life and received pirfenidone. At the time of the EIT measurement, the PFT showed a FVC of 45.5% pred. and DLco of 33.2% pred. The change in the end-expiratory lung impedance (dEELI) in this patient was already 71% above baseline at a PEEP level of 3 cm H_2_O, and further inversed to 126% at a PEEP level of 9 cm H_2_O. The medioventral and ventral parts of the lung were preferentially ventilated at the end of expiration (Figure 5, lowest panel).

**Figure 6:**
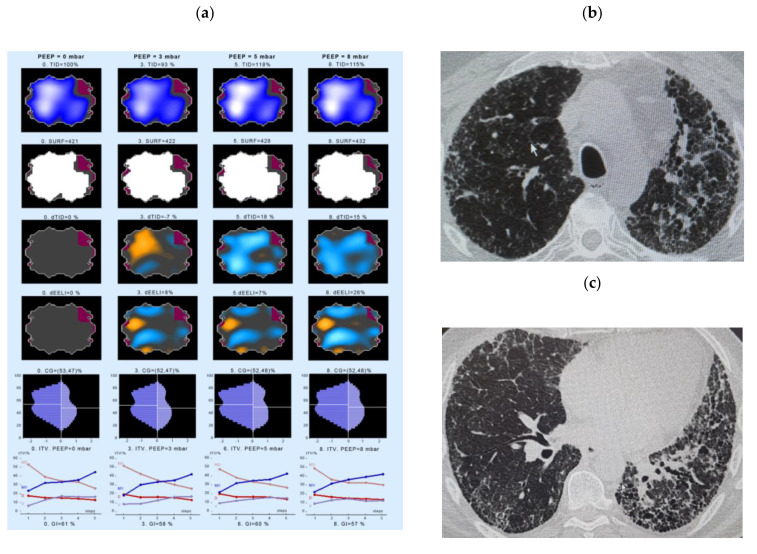
Changes in regional lung ventilation under increasing PEEP in IPF patient #12. Left panel (**a**): Spatial distribution of TID, SURF, dTID, dEELI in the EI tomogram at a PEEP levels of 0 (left), 3 (2nd left), 5 (3rd from left) and 8 cm H_2_O (right column). The lowest panel indicates the intratidal gas distribution (ITV) for four anatomical areas during one respiration. Given are the distribution of ventilation into the ventral (V), dorsal (D), medio-ventral (MV) and medio-dorsal (MD) regions of the lung. The X scale (1–5) shows the entire inspiration, divided into 5 steps. Right panel (**b**,**c**): The respective high resolution CT picture corresponding to the ROI of EIT measurement is shown. Abbreviations: CG—center of gravity of ventilation distribution; dEELI—difference of end-expiratory lung impedance over time; ITV—intratidal gas distribution; PEEP—positive end-expiratory pressure; SURF—surface of ventilated areas; TID—tidal impedance distribution; dTID—difference of TID over time.

Finally, we show the data of a 64-year-old IPF patient (ex-smoker, receiving pirfenidone). The PFT showed a FVC of 61.6% pred., Dlco of 32.2% pred. In this patient (Figure 6), the regional distribution of ventilation changed markedly during the first part of the inspiration, preferring lung areas in the right lower anterior parts of the lung (Figure 6, left panel, top line, TID). With increasing PEEP, the curves become flatter dorsally and ventrally. The change in end-expiratory lung impedance (dEELI) is small at the beginning and remains in this range at low PEEP levels before increasing to 26% at PEEP level of 8 cm H_2_O. In summary, EIT shows a stiff lung without change in lung volume as compared to baseline.

## 5. Discussion

In this study, we investigated if EIT could be of diagnostic help, serving as a radiation-free, non-invasive imaging tool for regional ventilation in spontaneously breathing IPF patients. Our research was driven on the observation that an altered pulmonary surfactant system in IPF leads to an increased alveolar surface tension and favors repetitive alveolar collapse. Assessment of the pulmonary ventilation, using real-time EIT imaging, was expected to potentially provide additional clues to this hypothesis by demonstrating regional ventilating abnormalities in IPF, caused by tissue scarring and by an increased surface tension. An increased surface tension could lead to increased alveolar collapse in IPF patients and this in turn could result in recruitment of alveoli when PEEP is increased.

Up to now, the main clinical benefit of EIT was the guidance in ventilator therapy by continuous monitoring of regional lung ventilation, aeration and respiratory system mechanics [17]. The previous publications from various EIT research groups, focusing on seeking optimal ventilator settings in experimental and human studies, demonstrated the usefulness of EIT monitoring in the respiratory management of patients with pulmonary conditions e.g., ARDS [18,30,31,32]. The study by Meier et al. used EIT to monitor the regional tidal volume during a PEEP titration maneuver in an experimental model of surfactant depletion. Based on the changes in regional ventilation secondary to the changes in the PEEP level, the researchers could identify the onset of collapse and regional lung recruitment even before global changes in pulmonary mechanics occurred [33].

Frerichs et al. examined the effect of exogenous surfactant and on the distribution of regional lung ventilation in newborn piglets with induced acute lung injury (ALI) [17]. They found that not only EIT reflected the ventilatory impairments, but also the combination of surfactant administration with PEEP recruitment significantly improved the ventral shift in ventilation distribution and asymmetry in the right-to-left lung ventilation distribution, additionally improving oxygenation and respiratory system compliance [17]. Lowhagen et al. showed 3 different distribution patterns in ALI and ARDS. In the first group, ventral and medio-ventral gas distribution decreased, and medio-dorsal and dorsal gas distribution increased at all PEEP stages during inspiration. In the second group, there was only a marginal difference in regional gas distribution, regardless of the PEEP level. A third pattern showed a marked increase in ventral distribution, while the medio-dorsal distribution decreased significantly at low PEEP levels and moderately decreased at high PEEP Levels [34]. In our study cohort, various distribution patterns could be documented, mostly reflecting fibrosing muster of IPF patients (subpleural, mostly dorso-basal).

Another research investigating ventilation distribution by EIT during assisted modes, with preservation of spontaneous breathing, has been demonstrated by Radke et al. [35]. Over-assisted ventilation by higher pressure support levels resulted in a shift of ventilation distribution to the non-dependent part in patients with mild or moderate ARDS. However, in patients with severe ARDS and an extreme respiratory effort, ventilation distribution was highly different between non-dependent and dependent regions, temporally and quantitatively [18]. Before performing PEEP increase, determination of recruitability is required when the lungs are suspected to be vulnerable to high inspiratory opening pressures. EIT knowingly has the potential to determine recruitability without applying excessive opening pressure by measuring the regional compliance during a PEEP trial. An additional technique to estimate lung recruitability was reported by Zhao and coworkers. They conducted research using a modified GI index and examined the relationship between the GI index and lung recruitability using a constant low-flow inflation maneuver in both ARDS and lung-healthy groups [28]. The modified GI index was calculated based on the differential EIT functional images obtained between different time points during prolonged inspiration [18,31]. In this line of reasoning, it is well known that alveolar recruitment in ARDS patients takes place during the entire inspiration and that, therefore, the inspiratory pressure, also has great impact on the process of recruitment, and not only the PEEP level.

This study provided a detailed analysis of EIT measurements in spontaneously breathing IPF patients vs. HC and found a significant increase in end-expiratory lung impedance (dEELI) values for both groups over the period of the PEEP increment, showing improved ventilation in recruited lung regions. When analyzing the regional changes in ventilation, we found a very inhomogeneous ventilation distribution in the IPF group, as reflected by the lower SURF values in the IPF versus the HC. The variables GI or CGVD were not significantly different between both groups; neither did these parameters change over time and in response to a PEEP increment.

As also evident from the individual trajectories, there were indeed IPF subjects responding well to the PEEP escalation protocol. Then, patients showed changes compatible with a recruitment of lung areas (one example shown in Figure 3). However, other IPF patients were not responding at all (e.g., presented in Figure 6). In more detail, an increase of dEELI was encountered in both groups at the end of the PEEP escalation protocol, suggestive of an improved ventilation or recruitment of lung regions and was found to be highly significant, when taking into account the entire course of the data for each group. Contrary to our expectations, however, there was no difference in dEELI between IPF patients and HC.

We are currently unable to explain the missing difference in dEELI between the IPF and the HC group. One possible explanation may be that the relative contribution of alveolar collapse, most likely occurring in the subpleural parts of the lungs, as compared to the overall increase in lung volume, may not be big enough to provoke significant differences between the IPF and the HC group.

The path forward for the EIT technology is the advantage of EIT being a real-time, radiation-free diagnostic instrument reflecting the regional ventilation and thus extent of periodic dystelectasis and atelectasis as well as (permanent) fibrotic changes in IPF. Following this line, the herein observed great variability in dEELI suggests that much higher numbers need to be investigated in future studies. Another improvement would be the combination of EIT with a lung function test, including the measurement of the transpulmonary pressure gradient via esophagus balloon. This would allow us to obtain EIT values throughout a standardized FRC maneuver and to relate EIT changes to intratidal and inter-individual transpulmonary pressure changes.

### Study Limitations

To the best of our knowledge, this is the first EIT study evaluating regional ventilation in an IPF cohort vs. healthy volunteers during spontaneous breathing. We aware of the following limitations:

- In general, due to spontaneous breathing, a profound heterogeneity and inconsistent variations were observed with regard to some EIT variables, which somewhat limited the overall conclusiveness. Also, not all IPF patients did manage to complete the protocol up to the highest PEEP level of 10, reporting that they felt uncomfortable and would experience the end-expiratory pressure as too high.
- Our control subjects were younger as compared to the IPF patients.
- Another limitation of our study was our inability to simultaneously assess EIT and lung function. Of our special interest would also be measurements of lung compliance and transpulmonary pressure gradients, which we would have liked to be correlated to the observed changes in regional ventilation.
- The results of this study were partly affected by the fact that subjects may react very differently to a PEEP titration. Here, the true transpulmonary pressure gradients (and hence the compliance) were unknown and, while some patients seemed to tolerate higher PEEP levels easily, others were not. Therefore, an optimal setting would be to combine the EIT measurement with a regular lung function test, including measurement of the transpulmonary pressure gradient via esophagus balloon and we expect this to be studied in the near future.

## 6. Conclusions

In this pilot study we could observe profound differences in regional ventilation and intratidal gas distribution in IPF patients. IPF subjects also showed a significant increase in the end-expiratory lung impedance in response to PEEP application, but there was no significant difference between spontaneously breathing IPF subjects and HC. To further evaluate how the disease severity and progression impact the EIT findings, we recommend the assessment of the EIT effects in the broader and larger cohort of different ILD phenotypes. Furthermore, we propose the combined measurements of EIT and lung function (under which EIT changes can be assigned to certain breathing maneuvers) in spontaneously breathing patients as a solution for a better comparability of the EIT variables.

### 6.1. Key Results

In some IPF patients we saw a significant increase in the end-expiratory lung impedance in response to PEEP application, but there was no significant difference to controls. We propose the combined measurements of EIT and lung function in the broader and larger cohort of different ILD phenotypes.

### 6.2. Summary Statement

Electric impedance tomography might be useful in the measurement of regional ventilation and gas distribution in IPF; further validation in larger cohort of different phenotypes of interstitial lung diseases is recommended.

## Figures and Tables

**Table 1:** Demographics and clinical parameters of our study cohorts.

Parameters	IPF (*n* = 17)	HC (*n* = 15)
Age (years) mean value ± SD (range)	65 ± 12 (44–79)	31 ± 14 (20–62)
Sex (male) *n* (% of the whole cohort)	13 (76%)	8 (53%)
Time to diagnosis (years) mean value ± SD (range)	3.1 ± 1.8 (0–7)	-
GAP Stage (I/II/III) *n* (% of the whole cohort)	6/9/2 (35%/53%/12%)	-
Body weight (kg) mean value ± SD (range)	80.9 ± 15.8 (46–109)	*n* = 11; 70.6 ± 17.3 (53–116)
BMI (kg/m2) mean value ± SD (range)	26.7 ± 4.1 (18.9–34.8)	24.4 ± 6.0 (19.4–39.2)
Smoker (active/never/ex) *n* (% of the whole cohort)	3/5/9 (18%/29%/53%)	-
Pack years mean value ± SD (range)	34 ± 20 (15–80)	-
Medication *n* (% of the whole cohort)	-	-
Prednisolone	7 (41%)	0 (0%)
Pirfenidone	8 (47%)	0 (0%)
N-Acetylcysteine	3 (18%)	0 (0%)
Azathioprine	3 (18%)	0 (0%)

Abbreviations: IPF—idiopathic pulmonary fibrosis; SD—standard deviation; GAP—Gender, Age, Physiology, BMI—Body Mass Index.

**Table 2:** Pulmonary function tests (PFT) values at measurement.

PFT Variable	IPF (*n* = 17) Mean Value ± SD (Range: Minimum–Maximum Value)	HC (*n* = 15) Mean Value ± SD (Range: Minimum–Maximum Value)
VC (% pred.)	72 ± 18 (44–114)	104 ± 10 (82–118)
FVC (% pred.)	71 ± 18 (46–118)	104 ± 11 (80–117)
TLC (% pred.)	77 ± 21 (50–126)	112 ± 12 (92–128)
FEV1 (% pred.)	76 ± 19 (47–132)	104 ± 10 (83–117)
FEV1/VC (% pred.)	106 ± 13 (77–134)	101.1 ± 5.1 (89–105)
ITGV (% pred.)	80 ± 27 (51–132)	102 ± 23 (66–140)
RV (% pred.)	92 ± 31 (60–160)	133 ± 35 (74–195)
RV/TLC (% pred.)	110 ± 12 (88–135)	116 ± 24 (69–151)
DLco (% pred.)	40 ± 16 (20–83)	87 ± 14 (71–120)
SaO_2_ (%)	95.3 ± 1.2 (92–97)	97.5 ± 0.9 (96–98)
pO_2_ (mmHg)	73.1 ± 7.6 (62–88)	89.2 ± 9.1 (77–103)
pCO_2_ (mmHg)	37.8 ± 3.4 (31–44)	37.2 ± 4.1 (31–44)

Abbreviations: DLco—diffusing capacity of the lung for carbon monoxide; FEV1—forced expiratory volume in one second; FEV1/VC—ratio of FEV1 to VC; FVC—forced vital capacity; ITGV—intrathoracic gas volume; pO_2_—partial pressure of oxygen; pCO_2_—partial pressure of carbon dioxide; RV—residual volume; TLC—total lung capacity; SaO_2_—oxygen saturation; SD—standard deviation; VC—vital capacity.

## Data Availability

Data is available upon request.

## Supplementary Materials

The following are available online at https://www.mdpi.com/2077-0383/10/2/192/s1, Material S1: Measured EIT variables in our study, Figure S1: Individual slope analysis of EIT variables (IPF vs. HC), Figure S2: Correlation of individual dEELI slopes with age, BMI, FVC (% predicted value) and weight for the whole cohort, Figure S3: Correlation of individual dEELI slopes with age, BMI, FVC (% predicted value) and weight in IPF patients, Figure S4: Correlation of individual dEELI slopes with age, BMI, FVC (% predicted value) and weight in HC, Table S1: Comparison of IPF and HC cohorts in regard to EIT values.
