# Supplementary material for: Evaluation of Regional Pulmonary Ventilation in Spontaneously Breathing Patients with Idiopathic Pulmonary Fibrosis (IPF) Employing Electrical Impedance Tomography (EIT): A Pilot Study from the European IPF Registry (eurIPFreg)
Source: J Clin Med. 2021 Jan 7;10(2):192. doi: 10.3390/jcm10020192 (PMC7827956; doi:10.3390/jcm10020192)
Supplement: Supplementary file 1 [file jcm-10-00192-s001.pdf]

Journal of
Clinical Medicine

MDPI

Supplementary Material S1: Measured EIT variables in our study.

1. **Intratidal gas distribution (ITV):** ITV, originally described by Loewhagen et al., exhibits the way the inspired gas distributes in the lungs during the tidal breath at defined ROIs. It therefore delineates the changes in regional ventilation with time course during one breath in different ROIs. When the lung region is divided into two ROIs, i.e. the ventral and dorsal regions, impedance changes from the beginning of inspiration to a certain time point in each region are expressed as a fraction of the contribution to global impedance change [30]. In our study, we have been analyzing 4 different ROIs, namely a ventral, a dorsal, a medioventral and a mediodorsal ROI.

   Tidal Impedance Distribution (TID): This parameter reflects the average ventilation for a defined period of breath, as reflected by the difference in impedance values at the beginning and the end of an inspiration. For the purpose of our study, we set the TID, as measured in IPF and controls at a PEEP level of zero, as 100%. For illustration of regional changes in TID, impedance changes above 10% of the maximum regional impedance change are displayed in dark blue. As values increase, the dark blue turns into a lighter blue. A white color indicates the regions of maximum regional impedance change
2. **Difference of TID over time (dTID):** This parameter reflects a global change of TID between two points of time, in our study the change in EELI between baseline (PEEP =0cm H_2O) and the various PEEP increment time points. dTID will be measured as % and as deviation to the reference section (dTID= TID-100) of the baseline value. For illustration of regional changes in dTID, the differential image indicates increases in dTID (vs. the reference) in turquoise and decreases in orange color.
3. **Surface of ventilated areas (SURF):** This parameter describes the surface of ventilated areas of the tomogram, as defined by a regional impedance chance between inspiration and expiration. A pixel whose regional impedance change exceeds 10 % of the maximum impedance change within the section is interpreted as ventilated. For global analysis, SURF is given as number of ventilated pixels for one section. For illustration of regional changes in the tomogram, ventilated pixels are displayed in white color; non-aerated pixels are given in a dark grey color.
4. **Global inhomogenity index (GI):** This parameter represents the spatial extent and dispersion in the distribution of tidal breath, i.e., global inhomogeneity in tidal ventilation [31]. For the global GI, this value is calculated as the sum of absolute differences between the median impedance value of all pixels and each single pixel value, divided by the sum of all impedance values in order to normalize the calculated values. The smaller the GI, the more homogeneous the tidal volume is distributed within the ventilated area. A GI with a value 0 represents a perfectly homogeneous distribution. For illustration of regional changes in the tomogram, the absolute differences between the median impedance value of all pixels and each single pixel value is depicted. No difference between the single pixel and the median value is depicted in white color, differences in are depicted in darker color in dependency of the magnitude of difference.
5. **Center of ventilation (CGVD):** The center of ventilation (CGVD), which was originally introduced by Frerichs et al., represents a vertical shift of the ventilation distribution along a gravitational axis in supine position [19]. CGVD (Center of Gravity of Ventilation Distribution) describes how ventilation is distributed between ventral and dorsal lung regions [32]. For the

global analysis, this variable is expressed as percentage of impedance on the dorsal-ventral scale from 0% (= ventilation completely in the dorsal image line) to 100% (in the ventral image line), representing the mean value of all TID values for each pixel. A value of 50 represents equally distributed ventilation between the ventral and the dorsal regions. Higher values represent that most of tidal ventilation is located in ventral regions (line is displayed towards ventral regions), and lower values indicate that most of tidal ventilation are located in dorsal regions (line shift toward dorsal regions). For illustration of regional changes in the tomogram the right and the left halves of ventilated area separately. Each half is divided in 32 equally spaced coronal slices. The sums of ventilation-related relative impedance changes are calculated for all pixels lying within the individual ROIs and the values are presented as two histograms, representing the right and the left lung, respectively.

6. **End-expiratory Lung Impedance (EELI):** This parameter reflects the impedance at end-expiration. The higher the EELI, the more lung volume is present at end-expiration. However, as the absolute lung volume is rarely known, EELI cannot be directly related to the end-expiratory lung volume. For the global analysis, EELI is set 100% for the baseline condition.
7. **Difference of EELI over time (dEELI):** This parameter reflects a change of EELI between two points of time; in our study the change in EELI between baseline (PEEP = 0 cm H_2O) and the various PEEP increment time points. For the global analysis, the differences of all sections are scaled to the reference section and are given in percentage of the baseline value. For illustration of regional changes, the dEELI value of each pixel is indicated by a blue to white color and the more color the higher the percental increase.

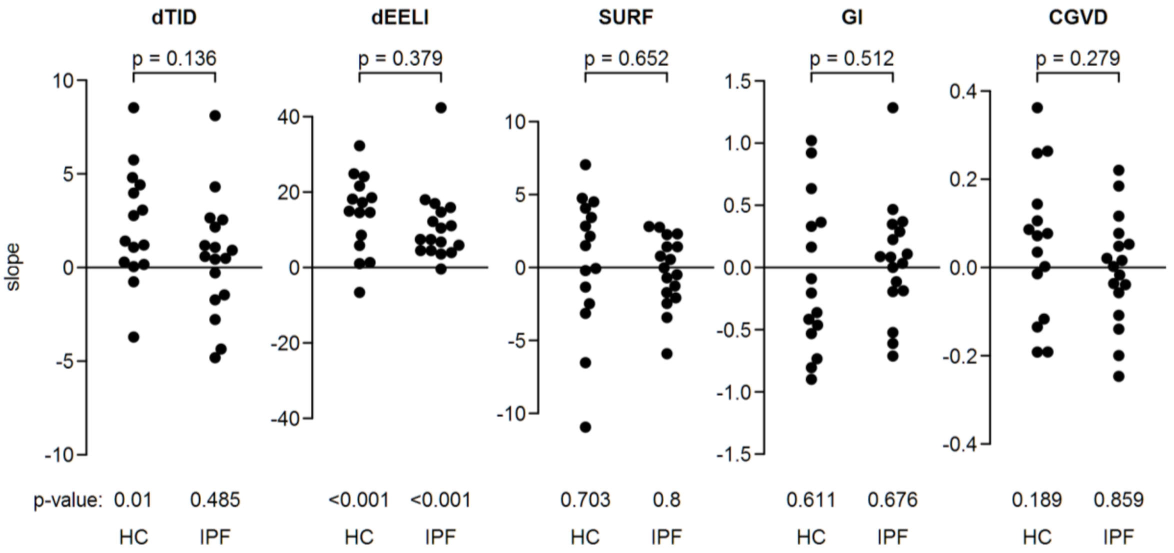

Supplementary Figure S1: Individual slope analysis of EIT variables (IPF vs. HC). Points show the slopes obtained from individual patient trajectories. Abbreviations: CGVD - center of gravity of ventilation distribution, dEELI- difference of end-expiratory lung impedance over time, GI - global inhomogenity index, PEEP- postivie end-expiratory pressure, SURF - surface of ventilated areas, TID- tidal impedance distribution, dTID- difference of TID over time.

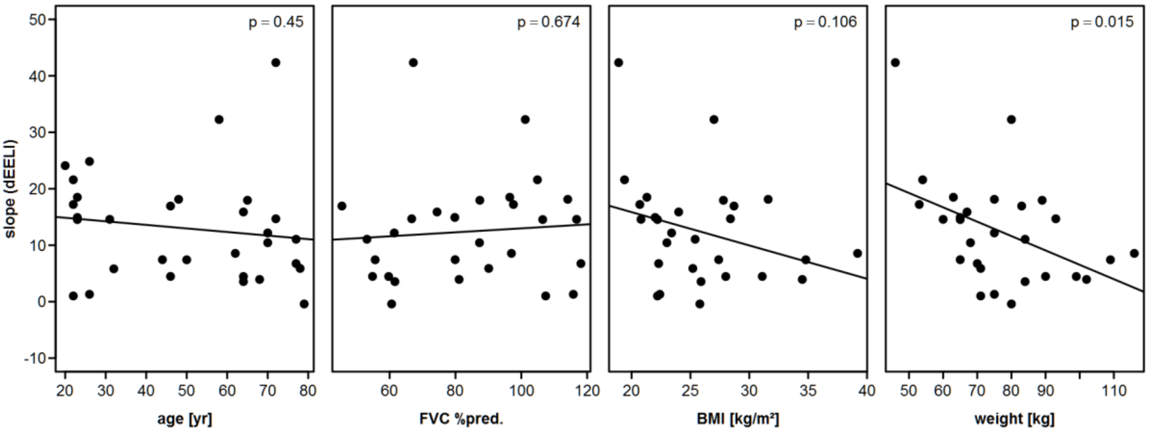

Supplementary Figure S2: Correlation of individual dEELI slopes with age, BMI, FVC (% predicted value) and weight for the whole cohort. Points represent data from individual patients. Solid line: regression line not adjusted for gender. Abbreviations: BMI- body mass index, FVC- forced vital capacity, % pred- percentage of predicted value.

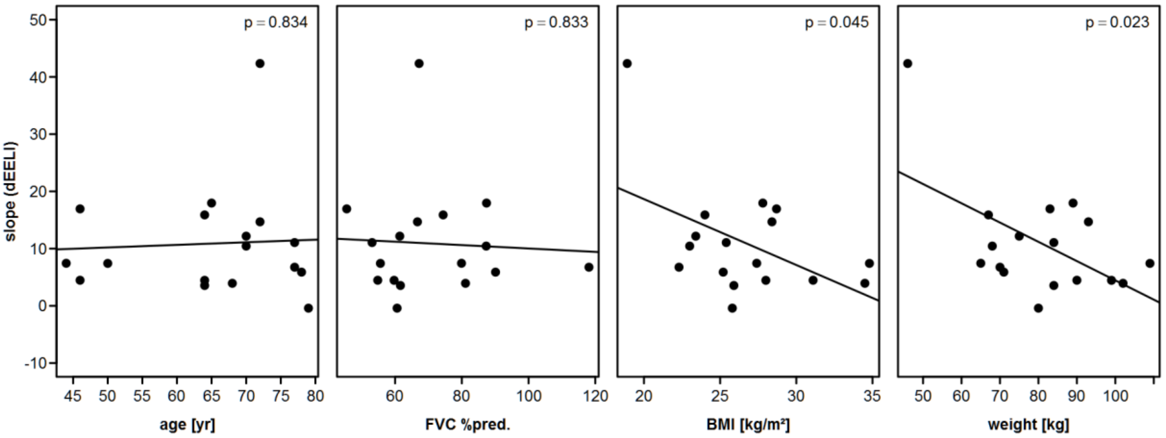

Supplementary Figure S3: Correlation of individual dEELI slopes with age, BMI, FVC (% predicted value) and weight in IPF patients. Points represent data from individual patients. Solid line: regression line not adjusted for gender. Abbreviations: BMI- body mass index, FVC- forced vital capacity, % pred- percentage of predicted value.

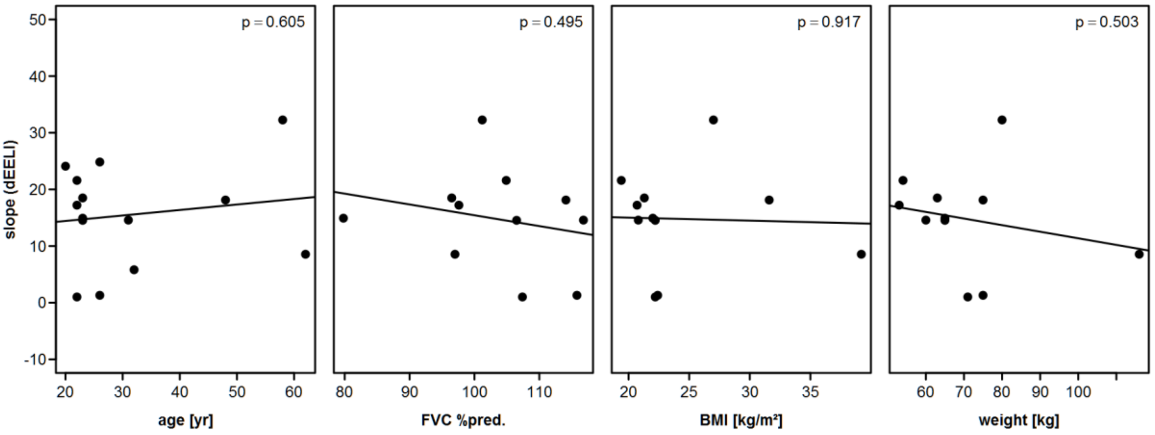

Supplementary Figure S4: Correlation of individual dEELI slopes with age, BMI, FVC (% predicted value) and weight in HC. Points represent data from individual patients. Solid line: regression line not adjusted for gender. Abbreviations: BMI- body mass index, FVC- forced vital capacity, % pred- percentage of predicted value.

Supplementary Table S1: Comparison of IPF and HC cohorts in regard to EIT values (given are point-wise mean values and 95% confidence intervals at each PEEP level).

	dTID			*dEELI*			*SURF*			*GI*			*CGVD*		
PEEP	*IPF*	*HC*	*IPF-HC*	*IPF*	*HC*	*IPF-HC*	*IPF*	*HC*	*IPF-HC*	*IPF*	*HC*	*IPF-HC*	*IPF*	*HC*	*IPF-HC*
0	0<br>(0, 0)	0<br>(0, 0)	0<br>(0, 0)	0<br>(0, 0)	0<br>(0, 0)	0<br>(0, 0)	404<br>(365, 444)	453<br>(406, 500)	-48.5<br>(-109, 12.3)<br>p = 0.114	56.5<br>(52, 61.1)	51<br>(45.6, 56.4)	5.55<br>(-1.5, 12.6)<br>p = 0.118	48.4<br>(46.8, 49.9)	47.7<br>(45.9, 49.5)	0.661<br>(-1.71, 3.03)<br>p = 0.571
1	5.97<br>(-3.2, 15.1)	-1.98<br>(-11.2, 7.2)	7.95<br>(-5.02, 20.9)<br>p = 0.216	10.8<br>(0.567, 21)	12.4<br>(2.16, 22.5)	-1.59<br>(-16, 12.8)<br>p = 0.82	421<br>(366, 476)	444<br>(389, 499)	-23.6<br>(-101, 54.2)<br>p = 0.534	54.7<br>(48.2, 61.2)	53.7<br>(47.2, 60.2)	0.978<br>(-8.24, 10.2)<br>p = 0.827	48.6<br>(46.5, 50.7)	47.5<br>(45.4, 49.6)	1.07<br>(-1.9, 4.04)<br>p = 0.462
2	6.1<br>(-2.01, 14.2)	2.81<br>(-5.83, 11.4)	3.3<br>(-8.55, 15.1)<br>p = 0.574	28.4<br>(9.69, 47.2)	17<br>(-2.92, 37)	11.4<br>(-16, 38.8)<br>p = 0.402	405<br>(366, 444)	448<br>(407, 490)	-42.8<br>(-99.7, 14.1)<br>p = 0.135	56<br>(51.1, 61)	52.9<br>(47.6, 58.2)	3.12<br>(-4.1, 10.3)<br>p = 0.385	48.6<br>(47.2, 50)	48.2<br>(46.7, 49.7)	0.389<br>(-1.68, 2.45)<br>p = 0.703
3	4.01<br>(-4.87, 12.9)	0.567<br>(-10, 11.1)	3.45<br>(-10.4, 17.3)<br>p = 0.613	33.7<br>(13.3, 54.2)	23.7<br>(-0.646, 48)	10.1<br>(-21.7, 41.8)<br>p = 0.522	405<br>(364, 447)	449<br>(400, 498)	-43.4<br>(-108, 20.9)<br>p = 0.177	56.4<br>(51.3, 61.6)	53.3<br>(47.2, 59.5)	3.09<br>(-4.91, 11.1)<br>p = 0.435	48.4<br>(47, 49.9)	47.7<br>(46, 49.5)	0.712<br>(-1.56, 2.99)<br>p = 0.526
4	6.94<br>(-4.97, 18.8)	3.82<br>(-8.48, 16.1)	3.12<br>(-14, 20.2)<br>p = 0.712	49.2<br>(17.8, 80.5)	42.9<br>(10.5, 75.3)	6.28<br>(-38.8, 51.3)<br>p = 0.778	407<br>(367, 446)	450<br>(409, 490)	-43<br>(-99.3, 13.3)<br>p = 0.129	56.5<br>(51.8, 61.2)	53.3<br>(48.5, 58.2)	3.14<br>(-3.64, 9.92)<br>p = 0.351	48.8<br>(47.3, 50.2)	48.2<br>(46.7, 49.7)	0.541<br>(-1.52, 2.6)<br>p = 0.595
5	6.27<br>(-6.21, 18.8)	7.84<br>(-5.06, 20.7)	-1.56<br>(-19.5, 16.4)<br>p = 0.86	60.1<br>(28.1, 92.1)	63<br>(30, 96.1)	-2.91<br>(-48.9, 43.1)<br>p = 0.898	404<br>(365, 442)	451<br>(411, 492)	-47.8<br>(-104, 7.96)<br>p = 0.09	56.6<br>(51.9, 61.3)	52.4<br>(47.6, 57.2)	4.21<br>(-2.5, 10.9)<br>p = 0.21	48.7<br>(47.3, 50.2)	48.2<br>(46.8, 49.7)	0.497<br>(-1.56, 2.55)<br>p = 0.625
6	3.71<br>(-7.82, 15.2)	11.9<br>(-1.78, 25.7)	-8.24<br>(-26.2, 9.69)<br>p = 0.354	68.3<br>(32.9, 104)	67<br>(24.9, 109)	1.28<br>(-53.7, 56.2)<br>p = 0.962	402<br>(366, 439)	461<br>(417, 505)	-58.3<br>(-116, -1.06)<br>p = 0.046	57<br>(52.1, 61.9)	50.9<br>(45, 56.7)	6.12<br>(-1.52, 13.8)<br>p = 0.112	48.4<br>(46.9, 49.9)	48.3<br>(46.6, 50.1)	0.0503<br>(-2.26, 2.36)<br>p = 0.965
7	8.18<br>(-3.48, 19.8)	18.7<br>(5.27, 32.2)	-10.6<br>(-28.4, 7.27)<br>p = 0.234	77.6<br>(36.2, 119)	81.1<br>(33.2, 129)	-3.46<br>(-66.8, 59.9)<br>p = 0.912	402<br>(365, 439)	459<br>(417, 502)	-57.4<br>(-113, -1.51)<br>p = 0.045	56.5<br>(51.9, 61.1)	51<br>(45.7, 56.2)	5.55<br>(-1.45, 12.5)<br>p = 0.115	48.3<br>(46.7, 49.9)	49<br>(47.1, 50.8)	-0.67<br>(-3.15, 1.8)<br>p = 0.583
8	9.39<br>(-3.67, 22.5)	14.4<br>(-1.36, 30.2)	-5.01<br>(-25.5, 15.5)<br>p = 0.619	89.8<br>(49.8, 130)	104<br>(55.5, 152)	-13.9<br>(-76.6, 48.7)<br>p = 0.651	411<br>(376, 446)	455<br>(413, 497)	-43.7<br>(-98.7, 11.3)<br>p = 0.114	55.7<br>(50.6, 60.9)	52<br>(45.7, 58.2)	3.78<br>(-4.3, 11.9)<br>p = 0.345	48.2<br>(46.7, 49.8)	48.3<br>(46.4, 50.2)	-0.0552<br>(-2.51, 2.4)<br>p = 0.963

Abbreviations: PEEP- positive end-expiratory pressure , SD- standard deviation, TID- distribution of ventilation , SURF- surface of ventilated area , dTID- delta of TID, dEELI- delta of end-expiratory lung impedance, GI- global inhomogeneity index, CG- center of gravity, n- patients number per PEEP grade.
